# LPA receptor 1 (LPAR1) is a novel interaction partner of Filamin A that promotes Filamin A phosphorylation, MRTF-A transcriptional activity and oncogene-induced senescence

**DOI:** 10.1038/s41389-022-00445-z

**Published:** 2022-12-28

**Authors:** Andreas Konopa, Melanie A. Meier, Miriam J. Franz, Emanuele Bernardinelli, Anna-Lena Voegele, Raja Atreya, Silvia Ribback, Stephanie Roessler, Achim Aigner, Kerstin Singer, Stephan Singer, Antonio Sarikas, Susanne Muehlich

**Affiliations:** 1 Department of Chemistry and Pharmacy, Molecular and Clinical Pharmacy, Friedrich-Alexander-Universität Erlangen-Nürnberg, Erlangen, Germany; 2 Institute of Pharmacology and Toxicology, Paracelsus Medical University, Salzburg, Austria; 3 Department of Medicine 1, Friedrich-Alexander-Universität Erlangen-Nürnberg, Erlangen, Germany; 4 Institute of Pathology, University of Greifswald, Greifswald, Germany; 5 Institute of Pathology, University of Heidelberg, Heidelberg, Germany; 6 Rudolf Boehm Institute of Pharmacology and Toxicology, Clinical Pharmacology, University of Leipzig, Leipzig, Germany; 7 Department for Pathology, University Hospital Tuebingen, 72076 Tuebingen, Germany

**Keywords:** Liver cancer, Target identification

## Abstract

Myocardin-related transcription factors A and B (MRTFs) are coactivators of Serum Response Factor (SRF), which controls fundamental biological processes such as cell growth, migration, and differentiation. MRTF and SRF transcriptional activity play an important role in hepatocellular carcinoma (HCC) growth, which represents the second leading cause of cancer-related mortality in humans worldwide. We, therefore, searched for druggable targets in HCC that regulate MRTF/SRF transcriptional activity and can be exploited therapeutically for HCC therapy. We identified the G protein-coupled lysophosphatidic acid receptor 1 (LPAR1) as a novel interaction partner of MRTF-A and Filamin A (FLNA) using fluorescence resonance energy transfer-(FRET) and proximity ligation assay (PLA) in vitro in HCC cells and in vivo in organoids. We found that LPAR1 promotes FLNA phosphorylation at S2152 which enhances the complex formation of FLNA and MRTF-A, actin polymerization, and MRTF transcriptional activity. Pharmacological blockade or depletion of LPAR1 prevents FLNA phosphorylation and complex formation with MRTF-A, resulting in reduced MRTF/SRF target gene expression and oncogene-induced senescence. Thus, inhibition of the LPAR1–FLNA–MRTF-A interaction represents a promising strategy for HCC therapy.

## Introduction

Myocardin-related transcription factors (MRTF-A/B, MKL1/2) are coactivators of the ubiquitously expressed transcription factor serum response factor (SRF) which controls fundamental biological processes such as cell growth, migration, differentiation, and cytoskeletal organization [1]. MRTFs convey stimulatory signals (e.g. lysophosphatidic acid (LPA) or serum stimulation) from RhoA to SRF via their translocation to the nucleus [2]. Emerging evidence reveals the importance of MRTFs and SRF in tumorigenesis [3]. Nuclear localization of MRTFs prevails in hepatocellular (HCC) and mammary carcinoma cells lacking the tumor suppressor deleted in liver cancer 1 (DLC1) [4]. Recent evidence implicates active nuclear MRTF-A as a dominant driver of tumor resistance and as a biomarker to predict tumor responsiveness to MRTF inhibitors [5]. Inhibitors of RhoA-stimulated MRTF/SRF gene transcription include CCG-1423 and its derivative CCG-222740, which reduce the activation of stellate cells in vitro and in vivo and thereby prevent the formation of stroma implicated in the pathogenesis of pancreatic cancer [6, 7]. We found that pharmacological inhibition of MRTF-A nuclear localization by the TRPM7 inhibitor NS8593 has antitumor effects by triggering oncogene-induced senescence [8]. Targeting MRTFs and their target genes Myoferlin (MYOF) and Tetraspanin 5 (TSPAN5) results in growth arrest mediated by oncogene-induced senescence in HCC cells and xenografts [9, 10]. Besides MYOF and TSPAN5, SM22 is aMRTF-A-dependent target gene [11] that was reported to be upregulated during gastric cancer progression and metastasis [12]. The metastatic potential of liver cancer stem cells (CSCs) arises from highly expressed SM22 [13]. The CSC concept states that tumor growth and metastasis are powered by a subset of tumor stem cells within HCCs [14]. Several lines of evidence suggest that the induction of oncogene-induced senescence (OIS) emerges as a key component for therapeutic intervention in HCC [3, 8]. Therefore, the identification of druggable targets that prevent MRTF/SRF transcriptional activity and induce oncogene-induced senescence in HCC is of utmost importance. Recently, we reported a novel mechanism of MRTF-A regulation through binding to the actin-binding protein Filamin A (FLNA) [15]. FLNA is susceptible to proteolysis and cleaved by calpain proteases to generate a 90 kDa C-terminal fragment [16]. The capability of calpain to cleave FLNA is regulated by the phosphorylation of FLNA. Phosphorylation at serine 2152 protects FLNA against cleavage [17]. Despite the importance of the MRTF-A–FLNA interaction for SRF transcriptional activity, the molecular mechanism of MRTF-A–FLNA complex formation remains unclear.

In this study, we report that lysophosphatidic acid receptor 1 (LPAR1)-mediated phosphorylation of FLNA at serine 2152 serves as the underlying molecular mechanism for MRTF-A–FLNA interaction, driving MRTF-A transcriptional activation and expression of MRTF/SRF target genes. Our results show for the first time, that LPAR1 is not only a key regulator of MRTF-A transcriptional activity in the cell but also a novel interaction partner of FLNA. LPAR1 is a class A rhodopsin-like GPCR with seven transmembrane helices that control multiple pathways leading to cell motility and cell growth [18]. LPAR1 is phylogenetically related to LPAR2 and 3, whereas LPAR4–6 are more closely related to the purinergic receptor family [19]. LPAR1–3 are phosphoproteins modulated by the natural agonist LPA, a bioactive phospholipid involved in the development but also in pathogenesis of fibrosis, inflammation, and cancer [20]. LPA has been identified as a driver of cell chemotaxis and invasion [21], and LPAR1 expression has been associated with increased invasiveness and metastasis in other cancer identities [22]. LPAR1 knockout mice show a reduction in both renal and pulmonary fibrosis [23, 24]. Several studies have suggested the potential value of LPAR1 inhibitors as a therapeutic strategy for lung fibrosis [25, 26].

Here, we report that depletion of LPAR1 inhibits MRTF/SRF target gene expression, resulting in the growth arrest of HCC cells due to oncogene-induced senescence. This is facilitated by the dissociation of the MRTF-A–FLNA complex and the redistribution of MRTF to the cytoplasm.

Our discovery that LPAR1 is overexpressed in HCC and the fact that GPCRs are highly druggable targets covering more than 40% of all drugs on the market [27] suggest that targeting LPAR1 is a promising, senescence-inducing therapeutic strategy to combat HCC growth.

## Results

### LPAR1 as a novel modulator of HCC cell proliferation and senescence

**Fig. 1:**
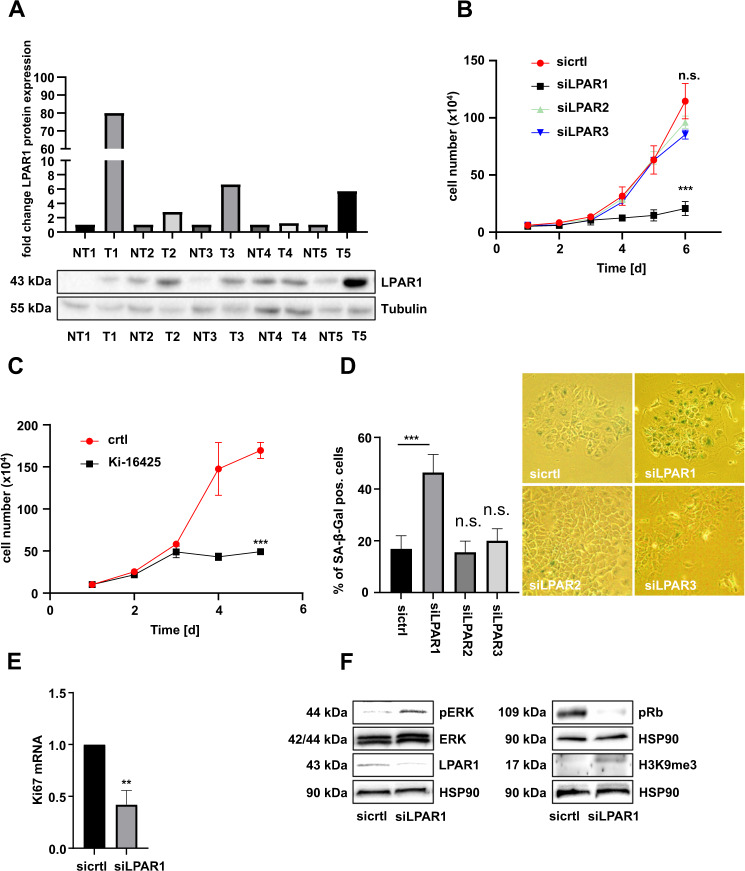
LPAR1 as a novel modulator of HCC cell proliferation and senescence. **A** Relative LPAR1 protein expression in human HCC tissue samples. Alternating non-tumorous (NT) and tumorous (T) samples, extracted from the same patients, were immunoblotted using anti-LPAR1 or anti-Tubulin antibody as a loading control and normalized to the non-tumorous sample. **B** HuH7 cells transiently transfected with LPAR1, LPAR2, and LPAR3 (siLPAR1, siLPAR2, and siLPAR3) siRNA or negative control (sicrtl), were counted daily for 6 days. Values are means ± SD (*n* = 3); ***p < 0.001. **C** HuH7 cells treated with or without Ki-16425 (20 µM) were counted daily for 5 days. Values are means ± SD (*n* = 3); ***p < 0.001. **D** Quantification of senescence-associated β-galactosidase (SA-β-gal) staining of HuH7 cells expressing LPAR1, LPAR2, and LPAR3 (siLPAR1, siLPAR2, and siLPAR3) siRNA and negative control siRNA (sicrtl) for 6 days (left). Representative pictures of SA-β-gal staining (right). Number of SA-β-gal positive blue cells was counted. Values are mean ± SD (*n* = 3); ***p < 0.001. **E** HuH7 cells transfected with negative control siRNA (sicrtl) or LPAR1 siRNA (siLPAR1) were subjected to qRT-PCR. Ki67 primers were used and normalization to the 18S rRNA was carried out. Values are means ± SD (*n* = 3); **p < 0.01. **F** Immunoblotting in lysates of HuH7 cells, harvested 6 days after transfection with negative control siRNA (sicrtl) or LPAR1 siRNA (siLPAR1) for pERK, ERK, LPAR1, and HSP90 (left), pRb, H3K9me3, and HSP90 (right).

Our previous studies demonstrated that pharmacological blockade or depletion of MRTFs or their target genes Myoferlin or Tetraspanin 5 inhibits HCC growth by inducing oncogene-induced senescence [3, 8, 10, 11]. We therefore searched for novel druggable targets at the plasma membrane overexpressed in human HCC patient samples that could constitute novel therapeutic targets to prevent MRTF function and HCC growth. We observed elevated expression of the G protein-coupled LPAR1 in tumorous tissue compared to non-tumorous tissue (Fig. 1A). Moreover, the HCC cell lines HuH6 and HuH7 exhibited strongly enhanced LPAR1 mRNA expression in contrast to the hepatoma cell line HepG2 (Fig. S1A). We next sought to decipher whether LPA receptor expression affects HCC cell proliferation by silencing LPA receptors using RNA interference and employing the commonly used LPAR1/3 inhibitor Ki-16425. LPAR1–3 siRNA efficiently reduced LPAR1–3 expression in HuH7 and HuH6 cells (Fig. S1B). Amongst these, only LPAR1 siRNA provoked a proliferation arrest in HuH7 and HuH6 HCC cells (Figs. 1B, S1C). A similar growth arrest was obtained upon administration of the LPAR 1/3 inhibitor Ki-16425 in HuH7 cells (Fig. 1C). We next tested whether the decrease in proliferation induced by LPAR1 depletion was caused by cellular senescence. There was a significant increase in senescence-associated ß-galactosidase (SA-ß-gal) positive HuH7 and HuH6 cells upon LPAR1 depletion (Figs. 1D and S1D), but not upon LPAR2 or LPAR3 depletion (Fig. 1D). The lower proliferation rate in LPAR1-depleted HuH7 and HuH6 cells was also reflected in a decreased *Ki-67* mRNA expression (Figs. 1E, S1E). We next investigated well-known markers of oncogene-induced senescence [28] and observed phosphorylation of ERK, hypophosphorylation of Retinoblastoma (Rb) protein and accumulation of histone H3 methylated on lysine 9 upon LPAR1 depletion in HuH7 cells (Fig. 1F). These data indicate that the LPAR1 is overexpressed in HCC and regulates the balance between HCC cell proliferation and oncogene-induced senescence.

### LPAR1 regulates MRTF-A localization and MRTF/SRF target gene expression

**Fig. 2:**
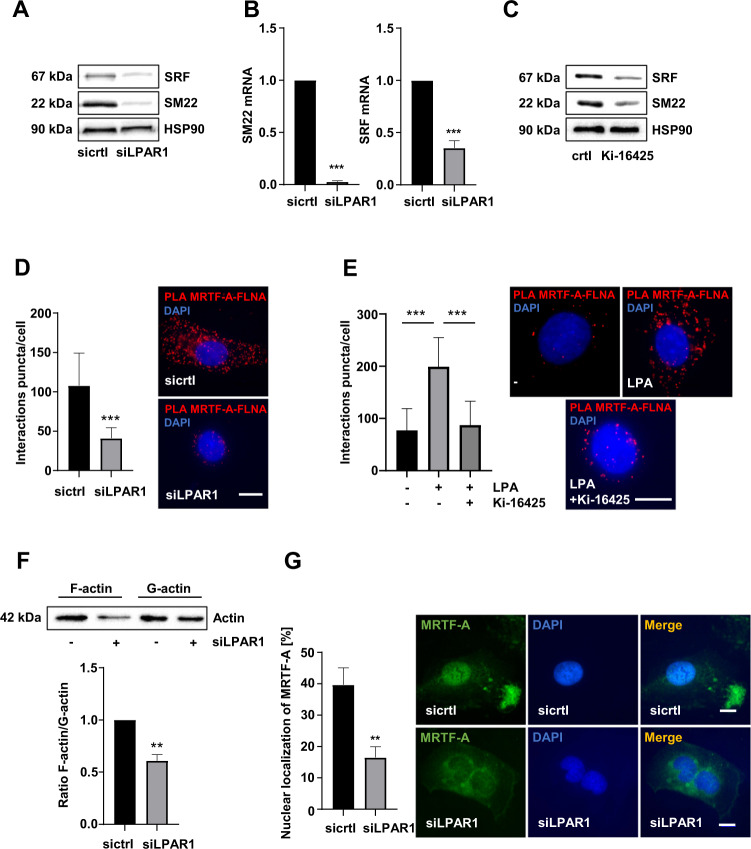
LPAR1 regulates MRTF-A localization and MRTF/SRF target gene expression. **A** HuH7 cells transiently transfected with LPAR1 (siLPAR1) and negative control (sicrtl) siRNA were subjected to immunoblotting for SM22, SRF, and HSP90 antibodies. **B** HuH7 cells treated as described above were subjected to qRT-PCR using *SM22* and *SRF* primers. Values are mean ± SD (*n* = 3); ***p < 0.001. **C** Immunoblotting for SRF, SM22, and HSP90 in lysates of HuH7 cells treated with or without Ki-16425 (20 µM). **D** Immunofluorescence analysis and quantification of proximity ligation assay for endogenous MRTF-A and FLNA in HuH7 cells expressing LPAR1 siRNA (siLPAR1) and negative control siRNA (sictrl). DAPI was used for nuclei staining. Scale bar: 10 µm. Values are mean ± SD (*n* = 3); ***p < 0.001. **E** Quantification of proximity ligation assay for endogenous MRTF-A and FLNA in HepG2 cells, which were untreated or treated with LPA (20 µM) for 30 min upon pre-incubation with or without Ki-16425 (20 µM) for 45 min. Scale bar: 10 µm. Values are mean ± SD; (*n* = 3); ***p < 0.001. **F** Immunoblotting of HuH7 cells transiently transfected with LPAR1 siRNA (siLPAR1) and negative control siRNA (sictrl) with anti-actin antibody (top). Quantification of the ratio of F/G-actin in HuH7 cells (bottom). Values are means ± SD (*n* = 3); **p < 0.01. **G** Statistical analysis of MRTF-A localization in HuH7 cells transfected with LPAR1 siRNA (siLPAR1) or negative control siRNA (sictrl) upon immunofluorescence staining with anti-MRTF-A antibody. Subcellular localization shown on the left was scored as predominantly nuclear in 100 cells per condition. Scale bar: 10 µm. Values are mean ± SD (*n* = 100 fields of vision) (*n* = 3); **p < 0.01.

We next tested whether LPAR1 regulates MRTF/SRF target genes and thereby oncogene-induced senescence. Knockdown of LPAR1 resulted in reduced expression of the established MRTF/SRF target genes *SM22* and *SRF* on protein as well as mRNA level (Figs. 2A and B, S2A). MRTF/SRF dependent target gene expression was also strongly decreased upon administration of the LPAR 1/3 inhibitor Ki-16425 in HuH7 cells (Fig. 2C). Based on our previous studies demonstrating an important role of MRTF-A interaction with FLNA for MRTF/SRF target gene expression [15], we reasoned that if LPAR1 regulates MRTF/SRF target gene expression, it should also affect MRTF-A–FLNA interaction. We used proximity ligation assays and found indeed abundant red puncta that were strongly decreased upon silencing of LPAR1 in HuH7 cells and LPAR1 blockade by the Ki-16425 inhibitor in LPA-treated HepG2 cells (Fig. 2D, E). In comparison to our previous studies in A7 melanoma cells which contain high levels of nuclear FLNA [15, 29], the FLNA content in HuH7 cells was equally distributed (Fig. S2B) and therefore an interaction between MRTF-A and FLNA detectable both in the cytoplasm and nucleus in HuH7 cells expressing control siRNA. In agreement with the requirement of F-actin for the MRTF-A–FLNA interaction [15], the cellular F-actin content was strongly decreased in HuH7 and HuH6 LPAR1 knockdown cells, as determined by actin fractionation and phalloidin staining (Figs. 2F, S2C, S2D). In order to analyze whether the decrease of F-actin upon LPAR1 depletion affected the nuclear localization of MRTF-A and thereby MRTF/SRF target gene expression, we depleted LPAR1 in HuH7 and HuH6 cells by RNA interference and followed the localization through immunofluorescence using an MRTF-A antibody. As a result, we observed a significant redistribution of MRTF-A to the cytoplasm in HuH7 and HuH6 cells upon LPAR1 depletion (Figs. 2G, S2E).

Taken together, our results implicate that LPAR1 plays a critical role in MRTF-A localization and MRTF/SRF target gene expression.

### LPAR1 mediates LPA-induced FLNA phosphorylation and MRTF-A–FLNA complex formation

**Fig. 3:**
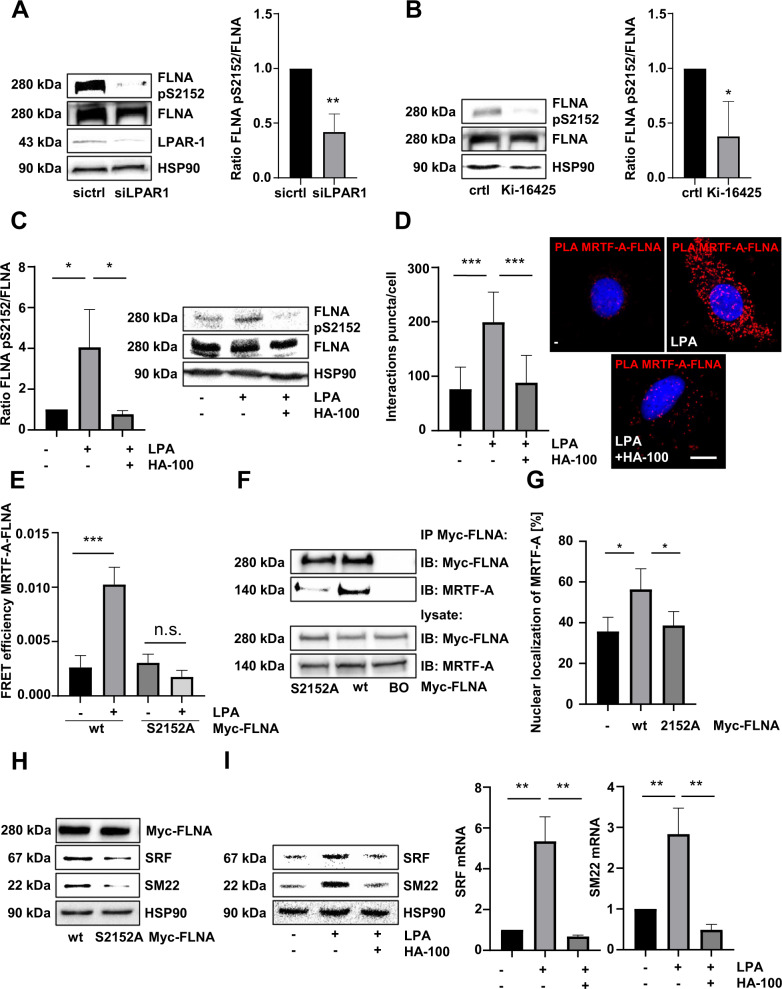
LPAR1 mediates LPA-induced FLNA phosphorylation and MRTF-A–FLNA complex formation. **A** Immunoblotting in lysates of HuH7 cells transfected with negative control siRNA (sictrl) or LPAR1 siRNA (siLPAR1), using anti-FLNA pS2152, anti-FLNA, anti-LPAR1, and anti-HSP90 antibodies (left). Quantification of the ratio of FLNA pS2152/FLNA (right). Values are means ± SD (*n* = 3); **p < 0.01. **B** Lysates of HuH7 cells treated with or without Ki-16425 (20 µM) were immunoblotted with anti-FLNA pS2152, anti-FLNA, and anti-HSP90 antibodies (left) and the ratio of FLNA pS2152/FLNA was quantified (right). Values are means ± SD (*n* = 3); *p < 0.05. **C** HepG2 cells were serum starved, pre-incubated with and without 10 µM HA-100 (10 µM) for 45 min, and then stimulated with 20 µM LPA for 30 min. Lysates were immunoblotted with anti-FLNA, anti-FLNA pS2152 and anti-HSP90 antibodies (right). Quantification of the ratio of FLNA pS2152/FLNA (left). **D** Immunofluorescence analysis and proximity ligation assay (PLA) quantification for endogenous MRTF-A and FLNA in serum-starved HepG2 cells, which were either untreated or treated with 20 µM LPA for 30 min upon pre-incubation with or without 10 µM HA-100 for 45 min. DAPI was used for nuclei staining. Scale bar: 10 µm. Values are mean ± SD (*n* = 3); ***p < 0.001. **E** FRET efficiency in serum-starved HepG2 cells transfected with Myc-FLNA or Myc-FLNA S2152A after stimulation with 20 µM LPA. Values are mean ± SEM (*n* = 3); ***p < 0.001. **F** Immunoprecipitation (IP) for Myc-FLNA and immunoblot (IB) for Myc-FLNA and MRTF-A in lysates from HuH7 cells expressing Myc-FLNA wt or Myc-FLNA S2152A. BO, Dynabeads-only control, without antibody. **G** Immunofluorescence analysis of MRTF-A localization in M2 cells transfected with empty vector (−), Myc-FLNA wt, and Myc-FLNA S2152A using anti-MRTF-A antibody and DAPI for nuclei staining. Values are mean ± SD (*n* = 100 fields of vision); (*n* = 3); *p < 0.05. **H** Immunoblotting for Myc, SRF, SM22, and HSP90 in lysates from M2 cells transfected with either Myc-FLNA wt or Myc-FLNA S2152A. **I** Immunoblotting for SRF, SM22, and HSP90 (left) in serum-starved HepG2 cells, pre-incubated with or without 10 µM HA-100 for 45 min and stimulated with or without 20 µM LPA. Quantitative real-time polymerase chain reaction (qRT-PCR) analysis of the mRNA expression of *SRF* and *SM22* in HepG2 cells after pre-incubation with or without 10 µM HA-100 and 4 h LPA stimulation (right). Values are mean ± SD (*n* = 3); **p < 0.01.

To decipher the molecular mechanism by which LPAR1 regulates MRTF/SRF target gene expression, we next examined the effect of exogenous LPA on the MRTF-A-FLNA interaction that promotes MRTF-A transcriptional activity [15]. Treatment of quiescent HepG2 cells with LPA induced FLNA phosphorylation at Ser 2152 within 15–90 min, determined by immunoblotting with an antibody that recognizes FLNA phosphorylated at Ser 2152 (Fig. S3A) [30]. The antibody did not react with the FLNA S2152A mutant in which the serine residue 2152 was mutated to alanine, confirming its specificity (Fig. S3B). We next examined whether the LPAR1 mediates FLNA phosphorylation. We observed strongly reduced FLNA phosphorylation at Ser 2152 in HuH7 and HuH6 LPAR1 knockdown cells (Figs. 3A, S3C), and upon administration of the LPAR1/3 inhibitor Ki-16425 (Fig. 3B). Consistent with previous studies showing the importance of PKC activity for FLNA phosphorylation, LPA-induced phosphorylation of FLNA at Ser 2152 was abrogated upon treatment with the PKA/C inhibitor HA-100 (Fig. 3C) [31]. To assess the importance of FLNA phosphorylation at Ser 2152 for MRTF-A–Filamin A complex formation, we used PLA and FRET assays. PLA assays showed abundant red puncta upon LPA stimulation that were almost completely abrogated in HepG2 cells preincubated with HA-100 (Fig. 3D). For FRET assays, MRTF-A tagged with cyan-fluorescent protein (CFP) and dsRed-tagged FLNA were co-transfected into HepG2 and HuH7 cells, with CFP and dsRed acting as FRET donor and FRET acceptor, respectively. MRTF-A-CFP gave a significant FRET signal with dsRed-FLNA-wt upon stimulation with LPA, which was abolished when MRTF-A-CFP was co-transfected with the non-phosphorylatable FLNA S2152A mutant (Figs. 3E, S3D). Likewise, the introduction of the FLNA S2152A mutant strongly reduced complex formation between MRTF-A and FLNA in coimmunoprecipitation assays in HuH7 cells expressing the FLNA S2152A mutant compared to wt FLNA (Fig. 3F). FLNA phosphorylation also regulated MRTF-A subcellular distribution. Expression of FLNA wt, but not the non-phosphorylatable FLNA S2152A variant, resulted in increased accumulation of MRTF-A in the nucleus of Filamin A-negative M2 cells (Figs. 3G and S3E). As MRTF-A localization and formation of the MRTF-A–FLNA complex is essential for the transcriptional activity of MRTF-A, we next assessed the importance of FLNA phosphorylation at Ser 2152 for the expression of MRTF/SRF target genes. Expression of the non-phosphorylatable FLNA S2152A mutant in FLNA-deficient M2 cells reduced the well-established MRTF target genes *SRF* and *SM22* compared to wt FLNA (Fig. 3H). Moreover, preincubation with HA-100 blocked LPA-induced expression of *SRF* and *SM22* both on mRNA and protein levels (Fig. 3I).

These results show that LPA-inducible FLNA phosphorylation at Ser 2152 promotes MRTF-A–FLNA interaction and MRTF/SRF target gene expression.

### Identification of LPAR1 as a novel interaction partner of FLNA and MRTF-A

**Fig. 4:**
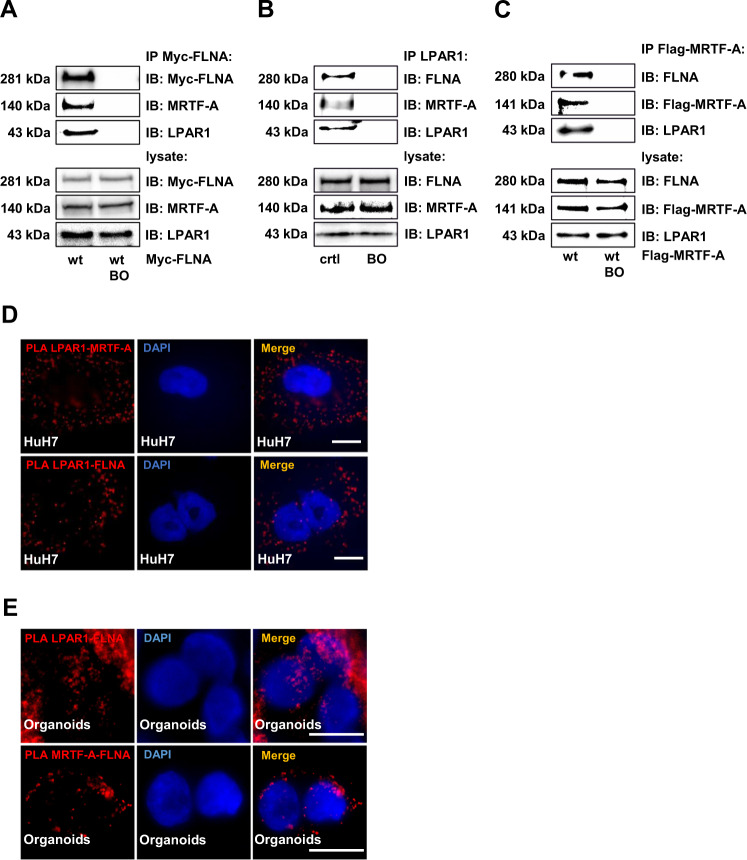
Identification of LPAR1 as a novel interaction partner of FLNA and MRTF-A. **A** Immunoprecipitation (IP) for Myc-FLNA in HuH7 cells expressing Myc-FLNA wt and immunoblot (IB) for Myc-FLNA, LPAR1, and MRTF-A. BO: Dynabeads-only control, without antibody. **B** Immunoprecipitation (IP) for endogenous LPAR1 in HuH7 cells and immunoblot (IB) for FLNA, MRTF-A, and LPAR1. BO: Dynabeads-only control, without antibody. **C** Immunoprecipitation (IP) for Flag-MRTF-A in HuH7 cells expressing Flag-MRTF-A. Anti-FLNA, anti-Flag-MRTF-A, and anti-LPAR1 antibodies were used for the immunoblot (IB). BO: Dynabeads-only control, without antibody. **D** Representative images of proximity ligation assay (PLA) for endogenous LPAR1 and MRTF-A (top) and LPAR1 and FLNA (bottom) signals in HuH7 cells. DAPI was used for nuclei staining. Scale bar: 10 µm. **E** Representative images of proximity ligation assay for endogenous LPAR1 and FLNA (top) and MRTF-A and FLNA (bottom) signals in organoids. DAPI was used for nuclei staining. Scale bar: 10 µm.

Data mining and biochemical experiments provided evidence that the helix 8 regions of a variety of GPCRs can bind to FLNA, and that this association is able to induce phosphorylation of FLNA by PKC [32]. We therefore tested whether the LPAR1 can bind to FLNA. We found a hitherto unknown association between LPAR1 and FLNA. LPAR1 was recovered from immunoprecipitates with Myc-antibody in HuH7 cells expressing Myc-FLNA (Fig. 4A), and FLNA from endogenous immunoprecipitations with LPAR1 (Fig. 4B). In agreement with our previous studies demonstrating a complex consisting of MRTF-A and FLNA [15], we additionally found an interaction between MRTF-A and LPAR1 in HuH7 HCC cells and M2 melanoma cells expressing FLNA (Figs. 4B and S4A). We confirmed the interaction of MRTF-A, FLNA, and LPAR1 by immunoprecipitation of HuH7 cells expressing FLAG-tagged MRTF-A. FLNA and LPAR1 were readily detectable after immunoprecipitation of FLAG-MRTF-A (Fig. 4C). We were also able to verify LPAR1 as a novel interaction partner of FLNA and MRTF-A in proximity ligation assays (Fig. 4D). To ultimately prove that the interactions occur in vivo, we performed PLA assays in human organoids. These revealed that LPAR1 interacts with FLNA and MRTF-A with FLNA in vivo (Fig. 4E).

### Mapping of LPAR1–FLNA interaction sites

**Fig. 5:**
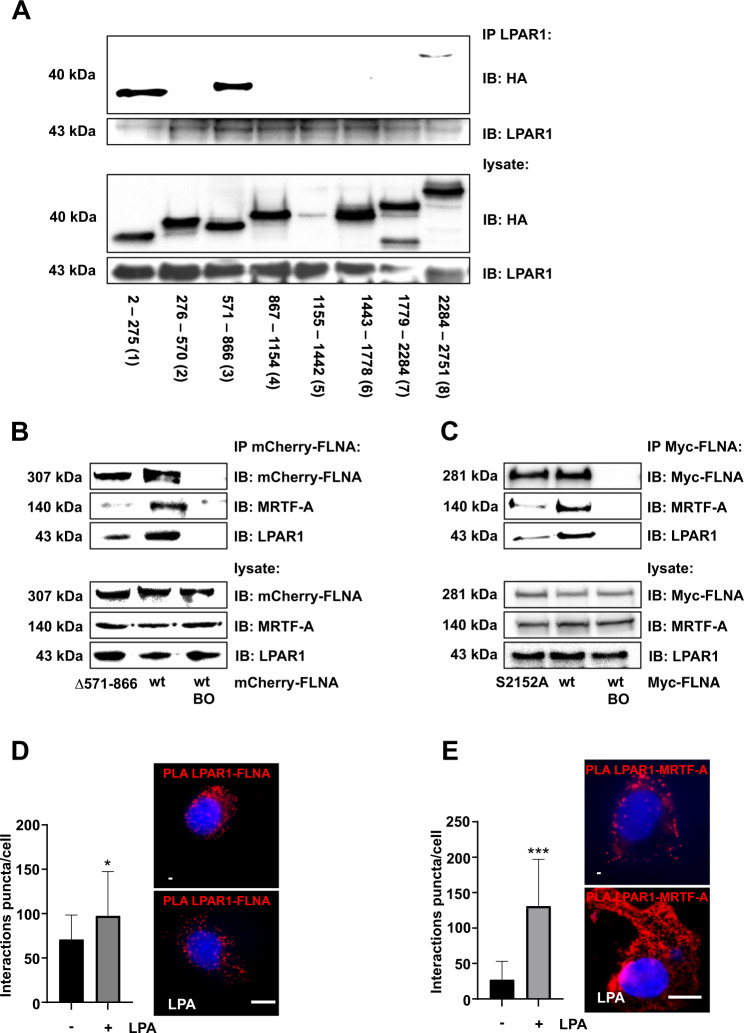
Mapping of LPAR1–FLNA interaction sites. **A** Immunoprecipitation (IP) in HuH7 cells transfected with different HA-FLNA fragments by using anti-LPAR1-antibody for pulldown and anti-LPAR1 and anti-HA antibodies for immunoblot (IB). HA-FLNA mutant 1-8: 1: FLNA a.a. 2-275; 2: FLNA a.a. 276-570; 3: FLNA a.a. 571-866; 4: FLNA a.a. 867-1154; 5: FLNA a.a. 1155-1442; 6: FLNA a.a. 1443-1778. 7: FLNA a.a. 1779-2284; 8: FLNA a.a. 2285-2751. **B** Immunoprecipitation (IP) for mCherry-FLNA in HuH7 cells expressing mCherry-FLNA or mCherry-FLNA Δ571-866 and immunoblot (IB) for mCherry-FLNA, MRTF-A, and LPAR1. BO: Dynabeads-only control, without antibody. **C** Immunoprecipitation (IP) for Myc-FLNA in HuH7 cells expressing Myc-FLNA or Myc-FLNA S2152A and immunoblot (IB) for Myc-FLNA, MRTF-A, and LPAR1. BO: Dynabeads-only control, without antibody. **D** Quantification of proximity ligation assay for endogenous LPAR1 and FLNA in HepG2 cells, which were stimulated with LPA (20 µM) for 30 min or remained untreated. Scale bar: 10 µm. Values are mean ± SD; (*n* = 3); *p < 0.05. **E** Quantification of proximity ligation assay for endogenous LPAR1 and MRTF-A in HepG2 cells, which were stimulated with LPA (20 µM) for 30 min or remained untreated. Scale bar: 10 µm. Values are mean ± SD; (*n* = 3); ***p < 0.001.

To map FLNA regions essential for the newly identified LPAR1 interaction, we used a series of hemagglutinin (HA)-tagged vectors spanning the FLNA protein in HuH7 cells. Immunoprecipitation of cell extracts with LPAR1 antibody revealed that the N-terminal actin-binding domain (FLNA-ABD; 2-275 (1)) and the MRTF-A-binding domain (FLNA 571-866 (3); [15]) of FLNA strongly interact with LPAR1 (Fig. 5A). In addition to these two prominent binding sites, FLNA has a third weaker binding site (FLNA 2284-2751 (8)) in the C-terminus. A similar 90 kDa C-terminal fragment is generated in HuH7 cells upon cleavage by calpain proteases and is expressed in HuH7 cells, as determined by calpain inhibitor III treatment (Fig. S4B). To further delineate the importance of the MRTF-A binding domain, we engineered an internal deletion of FLNA amino acids 571–866. Immunoprecipitation of HuH7 cell extracts with an antibody against the mCherry-tag and detection with mCherry-, MRTF-A- and LPAR1-antibodies proved that interaction with FLNA lacking amino acids 571–866 was strongly reduced for LPAR1 and MRTF-A (Fig. 5B).

Since other GPCRs such as Angiotensin II Typ 1 (AT1R) or the proto-oncogene MAS directly recruit FLNA and promote its phosphorylation at S2152 in an agonist-dependent manner [32], we next examined whether the FLNA S2152A mutant alters recruitment of LPAR1 and MRTF-A to FLNA. Indeed, an association of the FLNA S2152A mutant to LPAR1 and MRTF-A was markedly reduced (Fig. 5C). Likewise, LPA stimulation in HepG2 cells causing FLNA phosphorylation at S2152 elicited enhanced recruitment of FLNA and MRTF-A to LPAR1, as demonstrated by proximity ligation assays (Fig. 5D, E). Therefore, potential interaction sites with LPAR1 appear to be located in the N-terminal actin-binding domain, the MRTF-A binding domain, and the C-terminus of FLNA, and FLNA phosphorylation at S2152 promotes the binding of FLNA to the LPAR1 and MRTF-A.

### LPAR1 modulates cellular senescence and cytoskeletal alterations in concert with FLNA

**Fig. 6:**
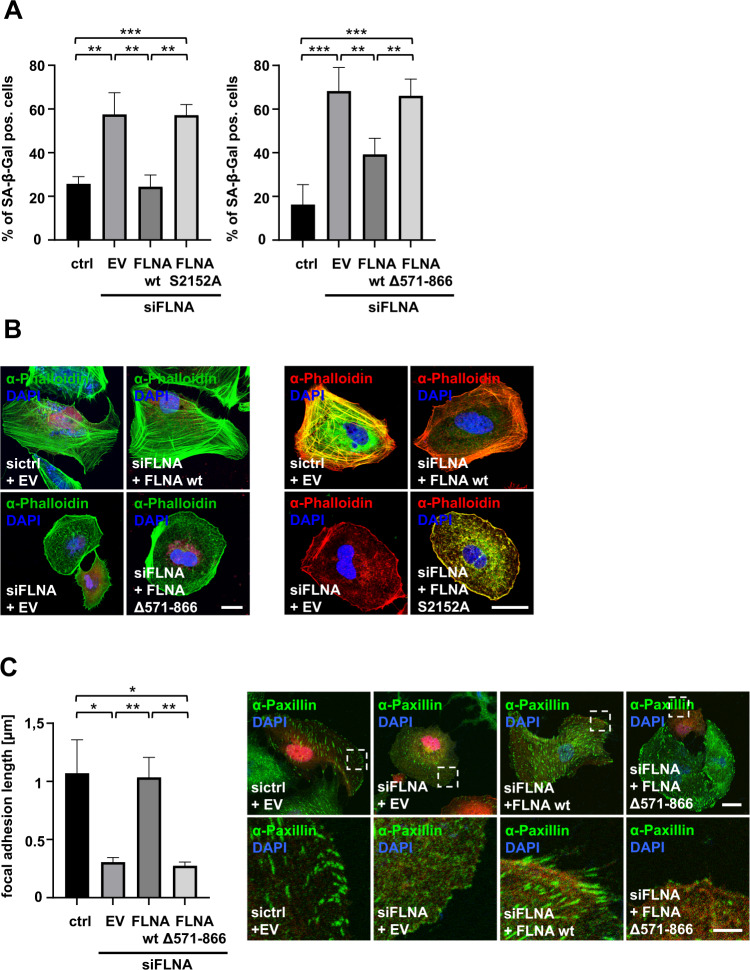
LPAR1 modulates cellular senescence and cytoskeletal alterations in concert with FLNA. **A** Quantification of senescence-associated β-galactosidase (SA-ß-gal) staining in HuH7 cells expressing negative control siRNA (ctrl) or FLNA siRNA (siFLNA), Myc-FLNA wt, Myc-FLNA S2152A or Myc-empty vector (EV) (left) or mCherry-FLNA, mCherry-FLNA Δ571-866 and –empty vector (EV) (right). A number of SA-β-gal positive blue cells was counted. Values are mean ± SD (*n* = 3); **p < 0.01, ***p < 0.001. **B** Immunostaining of F-actin by phalloidin in HuH7 cells transfected with scrambled siRNA (sictrl) or siFLNA and mCherry-FLNA (FLNA wt), mCherry-FLNA Δ571–866 (FLNA Δ571–866) or -empty vector (EV) (left) or FLNA siRNA (siFLNA), Myc-FLNA wt (FLNA wt), Myc-FLNA S2152A (FLNA S2152A) or -empty vector (EV) (right). DAPI was used for nuclei staining. Scale bar: 20 µm. **C** Quantification (left) of analysis of focal adhesion length by immunostaining (right) with anti-Paxillin antibody in HuH7 cells transfected with sictrl or siFLNA and mCherry-FLNA wt (FLNA wt), mCherry-FLNA Δ571-866 (FLNA Δ571–866) or -empty vector (EV). FLNA visualized in red. Scale bar: 20 µm. Values are mean ± SD (*n* = 3); *p < 0.05, **p < 0.01.

These latter results provided a structural framework for the newly identified LPAR1–Filamin A-MRTF-A axis, potentially regulating a variety of cellular responses such as the organization of the cytoskeleton and focal adhesions. Given the overexpression of LPAR1 in human HCC and the ability of LPAR1 depletion to induce oncogene-induced senescence (Fig. 1), we sought to analyze whether inhibition of the LPAR1–Filamin A interaction is sufficient for senescence induction, reorganization of the actin cytoskeleton and focal adhesions. SA-ß-gal staining revealed a strong increase in SA-ß-gal-positive cells upon FLNA knockdown using small interfering RNA (siRNA) against FLNA (Figs. 6A, S4C). Reintroduction of wt-FLNA, but not the non-phosphorylatable FLNA S2152A mutant or FLNA Δ571–866, which prevents association with LPAR1, counteracted senescence induction (Figs. 6A, S4D). Since senescence induction is accompanied by alterations in the cytoskeleton such as a decrease in actin stress fibers and the size of focal adhesions, we examined the actin cytoskeleton and focal adhesions by phalloidin and paxillin staining.

Immunofluorescence analyses in HuH7 cells transiently transfected with FLNA siRNA revealed decreased stress fiber formation (Fig. 6B). Re-expression of wt FLNA in FLNA-depleted cells, but not the Filamin A constructs defective in LPAR1 binding (FLNA Δ571–866 and FLNA S2152A) rescued stress fiber formation (Fig. 6B). Likewise, reduction of focal adhesion length in FLNA siRNA-expressing cells was prevented upon reconstitution of wt Filamin A, but not the Filamin A construct Δ571–866 (Fig. 6C). Knockdown efficiency of FLNA and uniform expression of FLNA wt and FLNA Δ571–866 was ensured by immunoblotting (Fig. S4D). Together, these results show that LPAR1 regulates in concert with FLNA senescence and senescence-associated alterations of the cytoskeleton.

## Discussion

In summary, we have established LPAR1 as a novel interaction partner of Filamin A and MRTF-A regulating MRTF/SRF target gene expression and oncogene-induced senescence. Since little progress has been made in HCC therapy and surgery remains the primary treatment of HCC, molecular insights into HCC formation and identification of novel targets for HCC therapy are urgently needed. In this paper, we provide the first evidence that LPAR1 is overexpressed in human HCC and that targeting LPAR1 results in growth arrest of HCC cells provoked by oncogene-induced senescence. Since senescence induction functions as a tumor-suppressive mechanism, strategies aimed at the induction of cellular senescence have gained considerable attention for HCC therapy [3]. We recently implicated MRTFs and their target genes Myoferlin and Tetraspanin 5 in the senescence response [10, 11], but the mechanisms driving the MRTF-associated senescence response remain unclear. Here, we show that LPAR1 activation facilitates FLNA phosphorylation at S2152, complex formation with MRTF-A, stress fiber- and focal adhesion formation, whereas impairment of FLNA phosphorylation caused by LPAR1 depletion or blockade redistributes MRTF-A to the cytoplasm and drives HCC cells into senescence. Mapping of the FLNA protein revealed that the N-terminal actin-binding domain (FLNA-ABD; amino acids 2–275) and the MRTF-A binding domain (FLNA amino acids 571–866) of FLNA are critical for the interaction with LPAR1. Besides the calponin homology (CH) domains including two actin-binding sites (ABS), FLNA-ABD is known to display a transmembrane domain which could play a crucial role in the interaction with LPAR1 [33]. In addition to these two prominent binding sites, FLNA has a third weaker C-terminal binding site (FLNA 2284–2751) for LPAR1. We found that a 90 kDa C-terminal fragment (FLNA CT) induced by calpain cleavage is naturally occurring in HuH7 HCC cells. Since FLNA CT has been shown to enhance the nuclear accumulation of multiple transcription factors such as HIF-1α, it is tempting to speculate that MRTFs transport to the nucleus may rely on this mechanism [34, 35].

**Fig. 7:**
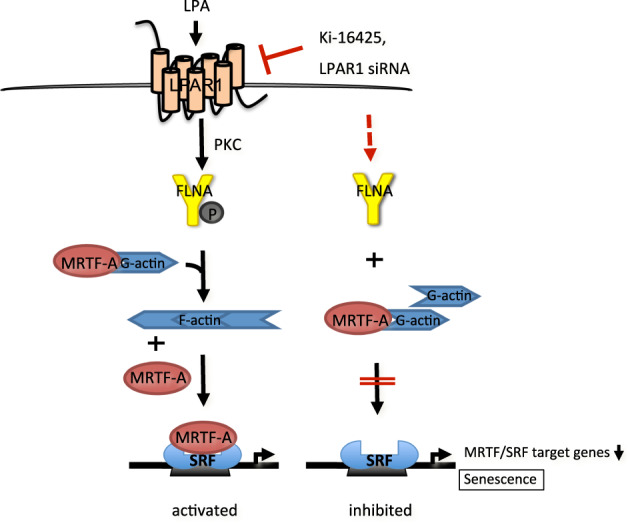
Model for LPAR1 regulation of FLNA and MRTF-A transcriptional activity. LPA induction leads to PKC activation and phosphorylation of FLNA at S2152, resulting in complex formation with MRTF-A and LPAR1 which triggers actin polymerization, resulting in dissociation of the repressive MRTF-A–G-actin complex and transcriptional activation of SRF. Depletion of LPAR1 inhibits FLNA phosphorylation at S2152, prevents complex formation with MRTF-A, inhibits MRTF/SRF target gene expression, and induces oncogene-induced senescence.

Comparative proteomics approaches correlated high FLNA expression with HCC progression, and FLNA phosphorylation at serine 2152 with high metastasis and poor prognosis [36, 37]. Our previous studies demonstrated that the MRTF-A–FLNA complex is important for MRTF/SRF transcriptional activity and cell migration [15]. In this study, we elucidate the mechanism of FLNA–MRTF-A complex formation and provide evidence that FLNA phosphorylation plays a crucial role in binding to LPAR1, actin polymerization, MRTF/SRF target gene expression, and senescence induction in HCC cells. In the model depicted in Fig. 7, LPA induction leads to PKC activation and phosphorylation of FLNA at S2152, promoting complex formation with MRTF-A and LPAR1, which triggers actin polymerization, resulting in dissociation of the repressive MRTF-A–G-actin complex and transcriptional activation of SRF. Depletion of LPAR1 inhibits FLNA phosphorylation at S2152, prevents complex formation with MRTF-A, inhibits MRTF/SRF target gene expression, and induces oncogene-induced senescence.

LPAR1 is a particularly exciting target for the treatment of HCC, as 80% of HCCs develop in the context of liver fibrosis. LPAR1 has been previously explored as a target against pulmonary fibrosis because mice with LPAR1 knockout exhibit reduced lung fibrosis following the bleomycin challenge [38].

There is evidence emerging that 7-transmembrane G-protein coupled receptors are rapidly internalized by endocytosis [39]. The actin nucleation-promoting protein N-WASP controls clathrin-mediated endocytosis and endosomal recycling of LPAR1, thereby driving tumor invasion and pancreatic cancer metastasis [40, 41]. Both clathrin and the adaptor protein ß-Arrestin target LPAR1 to clathrin-coated pits [42]. Interestingly, Filamin A also contributes to receptor recycling by controlling endosome motility, and its phosphorylation at Ser2152 is required to facilitate receptor recycling [43].

We propose a model in which LPAR1 signaling induces phosphorylation of FLNA at residue S2152 to stimulate LPAR1 endocytosis, and this phosphorylation event sustains receptor recycling to foster HCC cell proliferation and prevent senescence induction.

Taken together, our results open up the possibility that blocking the LPAR1 may be harnessed as a novel molecularly targeted therapeutic strategy for the treatment of HCC.

## Materials and methods

### Cell culture, transfections, and reagents

HuH7 and HepG2 cells were cultured in RPMI 1640 medium (Sigma-Aldrich, Taufkirchen, Germany), HuH6 cells in Dulbecco’s modified Eagle’s medium (DMEM; Sigma-Aldrich, Taufkirchen, Germany) and M2 cells in Minimum Essential Medium Eagle (MEM; Sigma-Aldrich, Taufkirchen, Germany) all supplemented with 10% fetal bovine serum (FBS; Invitrogen, Karlsruhe, Germany) and 1% Penicillin/Streptomycin (Sigma-Aldrich, Taufkirchen, Germany). For transient transfections of siRNA and plasmids lipofectamine RNAiMAX and lipofectamine 2000 (Invitrogen, Karlsruhe, Germany) were used according to the manufacturer’s instructions. The used siRNA sequences (Sigma-Aldrich, Taufkirchen, Germany, Horizon Discovery Cambridge, United Kingdom) and plasmids are listed in the supplemental information (Tables S1 and S2). For stimulation experiments, the cells were first serum starved in a medium supplemented with 0.2% FBS for 16 h and afterward treated with 20 µM LPA (Sigma-Aldrich, Taufkirchen, Germany). Inhibitor pretreatment with 10 µM HA-100 dihydrochloride (HA-100; Santa Cruz Biotechnology, Santa Cruz, CA, USA) or 20 µM Ki-16425 (Sigma-Aldrich, Taufkirchen, Germany) was performed for 45 min before addition of LPA.

### DNA cloning and plasmids

The mCherry-FLNA Δ571-866 deletion mutant lacking amino acids 571–866 was generated by using mCherry-FilaminA-N-9 (Addgene, Cambridge, MA, USA) and the primers listed in Supplementary Table 3. PECFP-MRTF-A was produced by sub-cloning the CDS of MRTF-A from p3xFLAG-MRTF-A. The S2152A point mutation was introduced in Myc-FLNA wt by site-directed mutagenesis with the QuickChange II Site-directed mutagenesis kit (Agilent, CA; USA).

### F-/G-Actin fractionation assay

Actin lysis buffer (50 m MMES pH 6.8, 50 mM KCl, 1 mM EGTA, 1 mM MgCl_2_, 0.5% Triton X-100 and protease inhibitor (Merck KGaA, Darmstadt, Germany)) was added to the cells to obtain the G-actin fraction before the cells were harvested and sonicated to receive the F-Actin fraction. Each fraction was subjected to immunoblotting using an anti-actin antibody (Sigma-Aldrich, Merck, Darmstadt, Germany).

### RNA extraction, cDNA synthesis, and quantitative real-time PCR analysis

For the mRNA isolation, TRIzol Reagent (Invitrogen, Karlsruhe, Germany) was used according to the manufacturer’s instructions. RNA was reverse transcribed into cDNA with SuperScript II Reverse Transcriptase (Invitrogen, Karlsruhe, Germany). Quantitative real-time PCR (qRT-PCR) was performed with SYBR Green I (Roche, Mannheim, Germany) and gene-specific primers (listed in Supplementary Table 4) at the LightCycler 96 system (Roche, Mannheim, Germany). For the normalization of target genes, the endogenous housekeeping control gene 18S rRNA was used.

### Cell proliferation assay

Cells were seeded and treated as described above. After the treatment, the number of cells was determined with a Neubauer counting chamber over a period of 5–6 days at intervals of 24 h.

### Immunoblotting

Proteins were mixed with Lämmli-buffer, boiled at 95 °C for 10 min, and separated according to their molecular weight by SDS–polyacrylamide gel electrophoresis (SDS–PAGE). Separated proteins were transferred onto a polyvinylidene fluoride (PVDF) membrane (Merck, Darmstadt, Germany), which was blocked with 5% milk powder in TBS-T followed by the incubation with the primary and the respective secondary antibodies listed in Tables S5 and S6. Blots were detected via chemiluminescence in a luminescent imager (ChemiDoc Imaging System, Bio-Rad Laboratories, Hercules, CA, USA).

### Senescence-associated ß-galactosidase staining

Cellular senescence was determined using the Senescence-ß-galactosidase staining kit according to the manufacturer’s instructions 5–6 days after treatment (Cell Signaling Technology, Danvers, MA, USA). By counting the number of blue cells per 100 cells in triplicates, the percentage of SA-β-gal-positive cells was calculated.

### In situ proximity ligation assay (PLA)

The DuoLink In situ Red Starter kits mouse/rabbit and goat/mouse (Sigma-Aldrich, Merck, Darmstadt, Germany) were used according to the manufacturer’s protocol for Duolink In situ solutions (Sigma-Aldrich, Merck, Darmstadt, Germany). Anti-MRTF-A (Santa Cruz Biotechnology, Santa Cruz, CA, USA), anti-FLNA (Millipore, Merck, Darmstadt, Germany), anti-LPAR-1 (Abcam, Cambridge, UK), and anti-LPAR-1 (Santa Cruz Biotechnology, Santa Cruz, CA, USA) antibodies were used as primary antibodies. Data were visualized with a confocal microscope (Carl Zeiss, Oberkochen, Germany) or a fluorescence microscope (Motic, Wetzlar, Germany), and data were analyzed by 5–15 randomly selected fields.

### Immunofluorescence (IF)

4% paraformaldehyde in PBS solution was used (10 min at RT) for the fixation of the cells, followed by permeabilization with 0.2% Triton X-100 in PBS for 7 min at RT. After blocking with 1% BSA in PBS for 30 min at 37 °C the cells were incubated with primary antibodies for 1 h at room temperature followed by the addition of the respective secondary antibodies labeled with Alexa Fluor 488/555/647 (Invitrogen, Karlsruhe, Germany). Used antibodies are listed in Tables S7/S8 in the supplementary material. F-actin filaments were stained with phalloidin coupled to Alexa Fluor 488/555 (Invitrogen, Karlsruhe, Germany), focal adhesions with anti-paxillin antibody, and nuclei with 4’,6-diamidino 2-phenylindole (DAPI; Sigma-Aldrich, Taufkirchen, Germany). Images were taken using a confocal microscope (Carl Zeiss, Oberkochen, Germany) and a fluorescence microscope (Motic, Wetzlar, Germany).

### FRET acceptor photobleaching fluorescence resonance energy transfer (FRET)

Cells were seeded on round 30 mm diameter glass slides and transiently transfected with the method of choice. 48 h after transfection media was changed to 0.2% FBS media and cells were starved for 18 h. After starvation, LPA was added to the media for a final concentration of 20 µM and cells were incubated for 45 min at 37 °C (control cells were incubated with water). After stimulation, cells were fixed for 30 min with 4% paraformaldehyde in Hanks’ Balanced Salt Solution (HBSS). Imaging was performed in HBSS at room temperature. The FRET acceptor dsRed was excited with a 514 nm Argon laser, and the emission was detected in the 525–650 nm range. The FRET donor ECFP was excited with a 405 nm laser, and the emission was detected in the 450–490 nm range. Imaging was performed by sequential acquisition with FRET AB-Wizard with a Leica TCS SP5II AOBS confocal microscope (Leica Microsystems, Wetzlar, Germany) equipped with an HCX PL APO ×63/1.20 Lambda blue water immersion objective and controlled by the LAS AF software (version 2.7.3.9723, Leica Microsystems). DsRed photobleaching in whole cells was obtained with 10 sequential illuminations at 514 nm (zoom factor 8×). FRET efficiency was calculated using the following formula:

$${{{\mathrm{FRET}}\,{\mathrm{efficiency}}}} = \frac{{1-{\mathrm{ECFPpre}}}}{{{\mathrm{ECFPpost}}}}$$

ECFPpre and ECFPpost refer to the ECFP intensity before and after dsRed photobleaching, respectively, and were determined in the entire bleaching region of interest (ROI).

### Co-Immunoprecipitation (CoIP)

Cells were lysed in 500 µl immunoprecipitation buffer (containing 0.2% phenylmethylsulfonyl fluoride, 150 mM NaCl, 50 mM Tris–HCl, protease inhibitor cocktail (Merck KGaA, Darmstadt, Germany) and 10% glycerol) and placed on ice for 45 min. Afterward, the samples were centrifuged at 12,700×*g* for 15 min at 4 °C. Meanwhile the Dynabeads (Invitrogen, Carlsbad, USA) were coupled to the respective antibodies. Used antibodies are listed in Table S9. 55 µl of the bead solution were coupled with 5 µl of the respective antibody by rotating for 30 min at RT and added to the supernatant of the lysed cells. The mixture was rotated overnight at 4 °C, washed with immunoprecipitation buffer, and subjected to sodium dodecyl sulfate–polyacrylamide gel electrophoresis according to the description above.

### Human liver biopsies

Tumorous and non-tumorous liver samples were extracted from routine surgical specimens respectively from the same patients and delivered to the Institute of Pathology, Greifswald Medical School. Ethical approval was received from the local Ethics Committee of the Greifswald Medical School (no. BB67/10).

### Nuclear and cytoplasmic fractionation assay

Cells were harvested with trypsin and the NE-PER Nuclear and Cytoplasmic Extraction kit was used according to the manufacturer’s protocol (Thermo Scientific, Waltham, USA).

### Organoid culture

Biopsies were washed three times with cold PBS and incubated with 2 mM EDTA in PBS for 30 min at 4 °C on a rotating wheel. The supernatant was restored with fresh, cold PBS, centrifuged (200×*g*, 5 min, 4 °C), and resuspended in Matrigel (Corning, New York, USA). Then 250 µl medium (Organoid Growth Medium, Stem cell, Vancouver, Canada) was added and organoids were cultured at 37 °C in a 5% CO_2_ atmosphere. The medium was changed every 2–3 days and organoids were passaged once a week. To obtain organoid monolayers for the PLA assay, the organoid medium was removed, and replaced with Cell recovery solution (Corning, New York, USA), and the plate was put on ice for 20 min to dissolve the Matrigel. The organoids were collected with cold PBS in a 15 mL tube and centrifugated at 200×*g* for 5 min at 4 °C. The pellet was washed with 5 ml cold PBS and again centrifugated, resuspended in 500 µl TripLE Express (Thermo Scientific, Waltham, USA), and incubated at 37 °C for 15 min. Organoids were dissociated into single cells by pipetting vigorously. The single cells were centrifuged at 200×*g* for 5 min, 4 °C. The pellet was resuspended in organoid medium, and cells were seeded into precoated (Matrigel, PBS 1:50; 1 h; 37 °C) chamber slides (Thermo Scientific, Waltham, USA).

### Statistical analysis

Statistical analysis was carried out using Student’s *t*-test (two-sided) or ANOVA. Unless otherwise indicated, data from three independent experiments were analyzed and values were presented as mean and standard deviation (mean ± SD). Values are considered significant with *p < 0.05, **p < 0.01, and ***p < 0.001.

## Supplementary information

Figure S1

Figure S2

Figure S3

Figure S4

Supplementary legends

Supplementary tables

## Data Availability

The authors confirm that the data supporting the findings of this study are available within the article and its supplementary materials.
