# Supplementary figures and images for: LPA receptor 1 (LPAR1) is a novel interaction partner of Filamin A that promotes Filamin A phosphorylation, MRTF-A transcriptional activity and oncogene-induced senescence
Source: Oncogenesis. 2022 Dec 28;11(1):69. doi: 10.1038/s41389-022-00445-z (PMC9797565; doi:10.1038/s41389-022-00445-z)

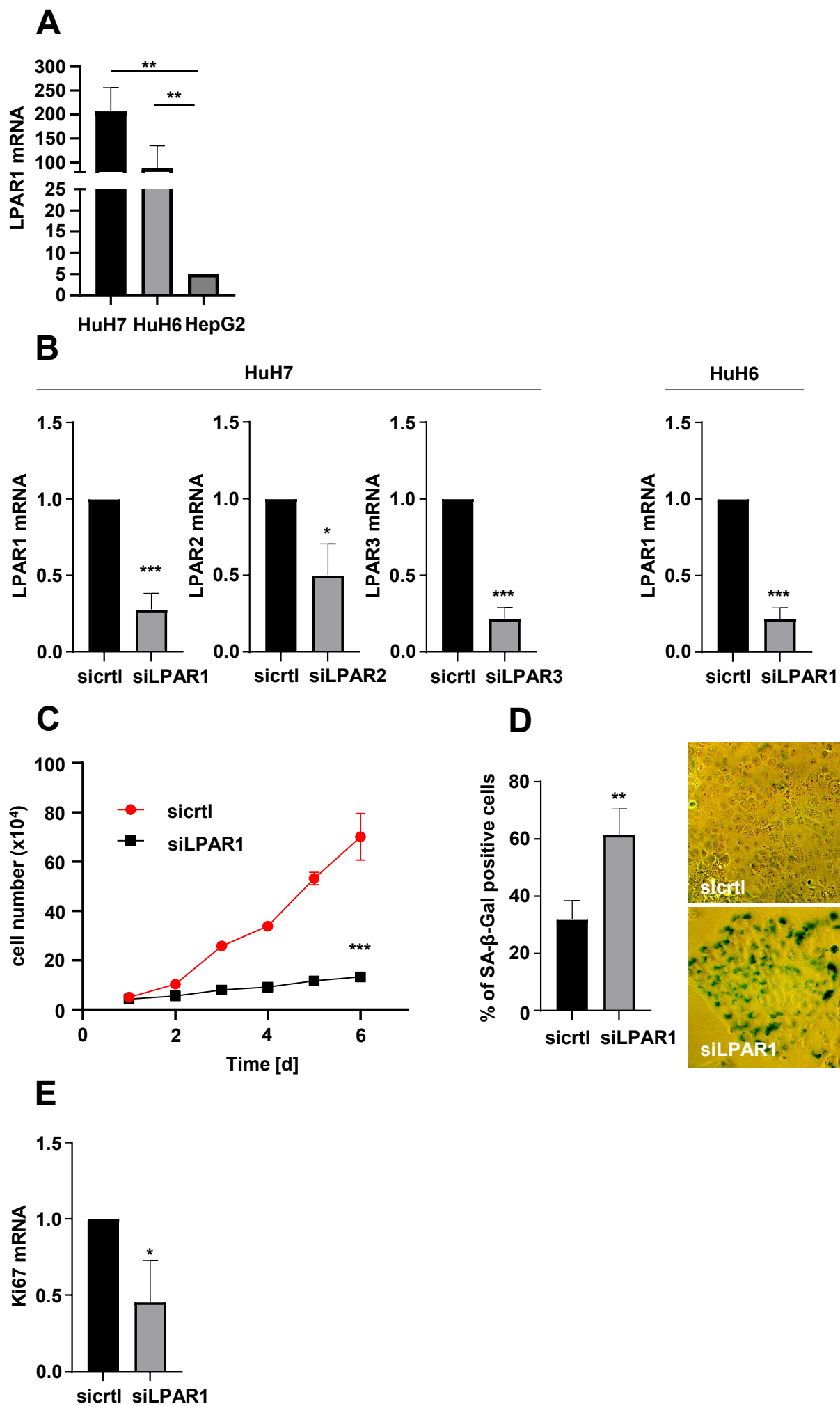

Fig. S1

Supplement: Supplementary file 1 — Figure S1 [file 41389_2022_445_MOESM1_ESM.pdf]

**A**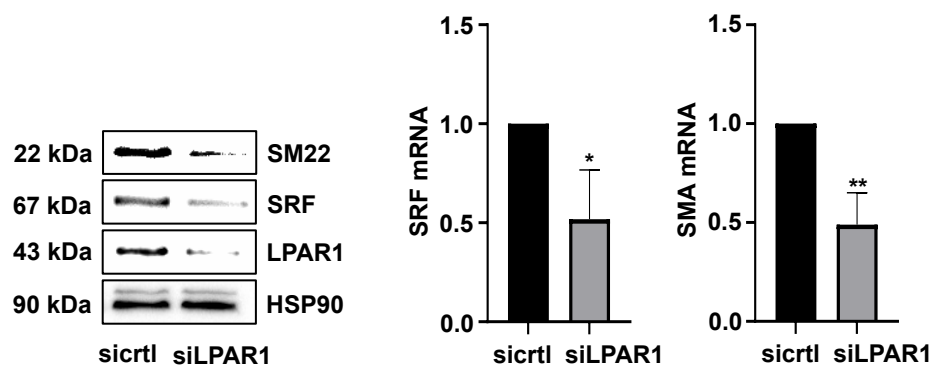**B**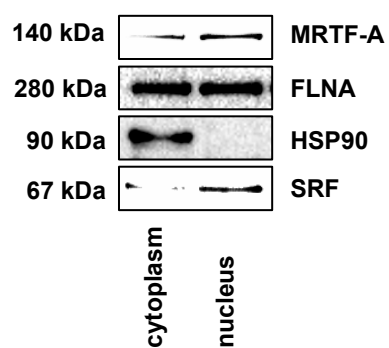**C**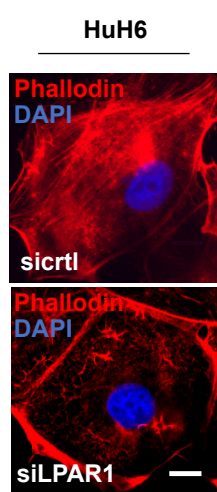**D**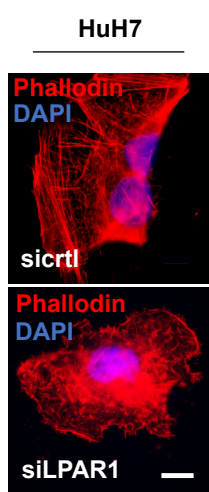**E**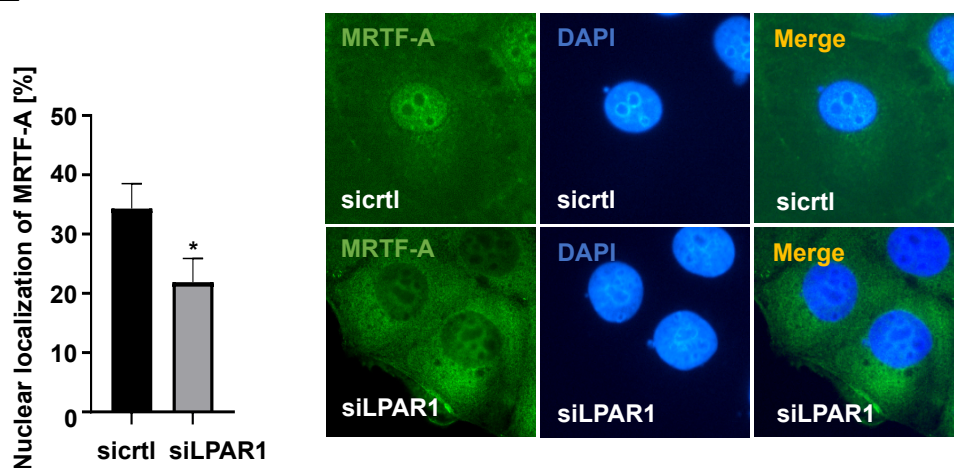**Fig. S2**

Supplement: Supplementary file 2 — Figure S2 [file 41389_2022_445_MOESM2_ESM.pdf]

**A**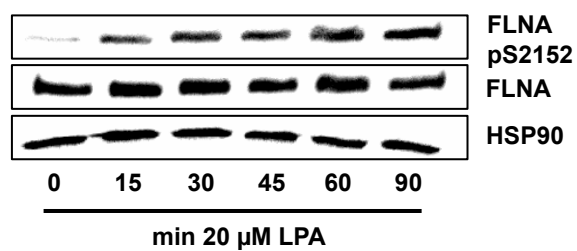**B**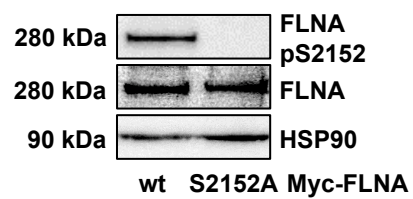**C**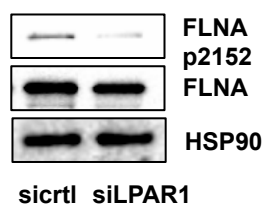**D**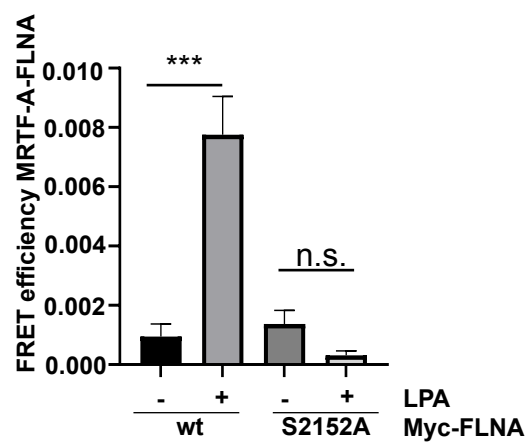**Fig. S3**

Supplement: Supplementary file 3 — Figure S3 [file 41389_2022_445_MOESM3_ESM.pdf]

**A**

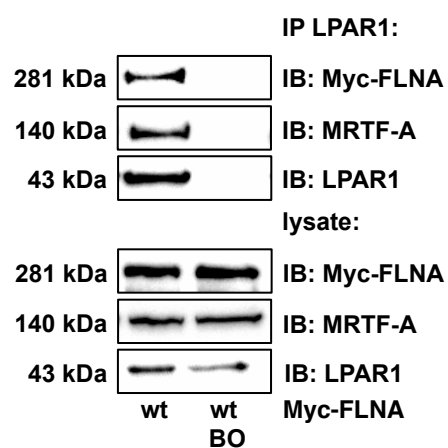

**B**

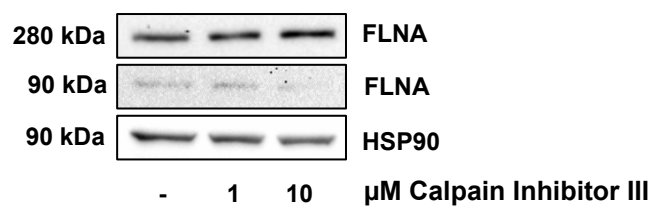

**C**

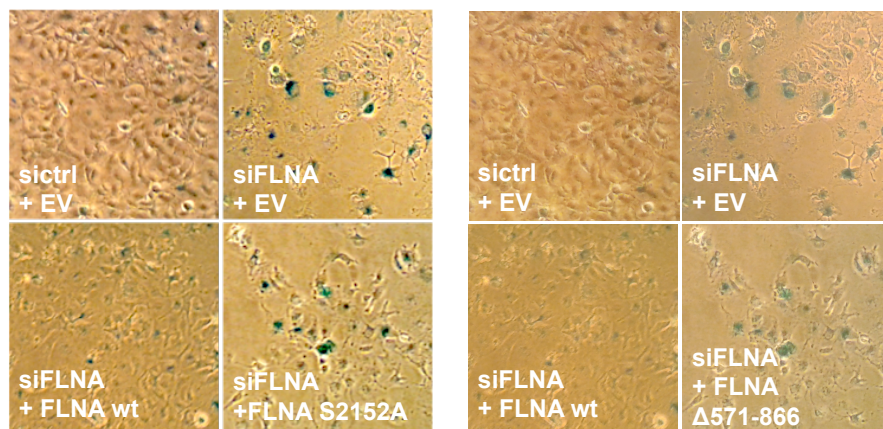

**D**

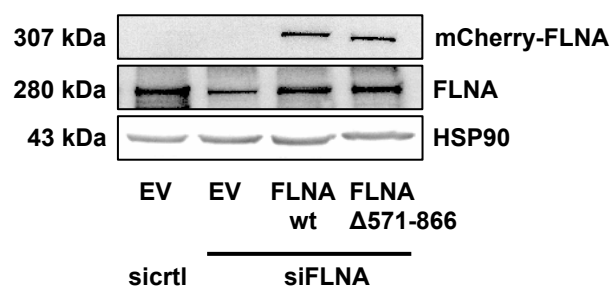

**Fig. S4**

Supplement: Supplementary file 4 — Figure S4 [file 41389_2022_445_MOESM4_ESM.pdf]
