# Supplementary material for: LPA receptor 1 (LPAR1) is a novel interaction partner of Filamin A that promotes Filamin A phosphorylation, MRTF-A transcriptional activity and oncogene-induced senescence
Source: Oncogenesis. 2022 Dec 28;11(1):69. doi: 10.1038/s41389-022-00445-z (PMC9797565; doi:10.1038/s41389-022-00445-z)
Supplement: Supplementary file 5 — Supplementary legends [file 41389_2022_445_MOESM5_ESM.docx]

**Supplementary Figures**

(S1A) LPAR1 mRNA expression of HuH7, HuH6 and HepG2 cells was assessed by qRT-PCR. LPAR1 primer was used and normalization to the 18 S rRNA was carried out. Values are means ± SD (n = 3); **p<0.01.

(S1B**)** LPAR1-3 mRNA expression in HuH7 (left) and LPAR1 mRNA expression in HuH6 cells (right) transfected with negative control siRNA (sictrl) or LPAR1-3 siRNA (siLPAR1-3) was assessed by qRT-PCR. The respective LPAR1-3-specific primers were used and normalization to the 18 S rRNA was carried out. Values are means ± SD (n = 3); *p<0.05; ***p<0.001.

(S1C) HuH6 cells transfected with negative control siRNA (sictrl) and LPAR1 siRNA (siLPAR1) were counted every 24 hours for 6 days. Values are means ± SD (n = 3); ***p<0.001.

(S1D) Quantification of senescence associated SA-β-galactosidase staining of HuH6 cells transfected with negative control (sicrtl) and LPAR1 siRNA (siLPAR1) (left). Representative pictures of SA‑β‑gal staining (right). Values are mean ± SD (n=3); **p<0.01.
(S1E**)** Ki67 mRNA expression upon transient knockdown using LPAR1 siRNA (siLPAR1) for 4 days compared with negative control siRNA (sicrtl) in HuH6 cells was assessed by qRT-PCR. The respective gene-specific primer was used and normalization to the 18 S rRNA was carried out. Values are means ± SD (n = 3); *p<0.05.

(S2A) HuH6 cells transfected with LPAR1 siRNA (siLPAR1), or negative control siRNA (sicrtl) were immunoblotted for SRF, SM22, LPAR1 and HSP90 (left) or analyzed by qRT-PCR with the primers form SRF and SMA. Values are means ± SD (n = 3); *p<0.05.

(S2B) Immunoblot of nuclear and cytoplasmic fractions of HuH7 cells using MRTF-A, FLNA, HSP90 (control for the cytoplasmic fraction) and SRF (control for the nuclear fraction) antibodies.

(S2C) The actin cytoskeleton of HuH6 cells transfected with scrambled siRNA (sictrl) or siLPAR1 (siLPAR1) was visualized after Alexa Fluor 488 phalloidin binding by immunofluorescence analysis. DAPI was used for nuclei staining. Scale bar: 10 µm.

(S2D) Visualization of the actin cytoskeleton of HuH7 cells transfected with negative control siRNA (sictrl) or LPAR1 siRNA (siLPAR1) by binding of Alexa Fluor 488 phalloidin. DAPI was used for nuclei staining. Scale bar: 10 µm.

(S2E) Statistical analysis of MRTF-A nuclear localization in HuH6 cells transfected with LPAR1 siRNA (siLPAR1) or negative control siRNA (sictrl) upon immunofluorescence staining with anti-MRTF-A antibody. Subcellular localization shown on the right was scored as predominantly nuclear in 100 cells per condition. Scale bar: 10 µm. Values are mean ± SD (n=100 fields of vision); (n = 3); *p<0.05.

(S3A**)** Quiescent HepG2 cells were stimulated as indicated with 20 µM LPA prior to lysis and immunoblotted with anti-FLNA, anti-FLNA pS2152 and anti-HSP90 antibody as a loading control.

(S3B) M2 cells transfected with Myc-FLNA wt or Myc-FLNA S2152A were immunoblotted with anti-FLNA, anti-FLNA pS2152 and anti-HSP90 antibodies.

(S3C) Immunoblotting in lysates of HuH6 cells transfected with scrambled siRNA (sictrl) or siLPAR1 using anti-FLNA p2152, anti-FLNA and anti-HSP90 antibodies. (S3D) FRET Efficiency in serum starved HuH7 cells transfected with Myc-FLNA or Myc-FLNA S2152A after stimulation with 20 µM LPA; Values are mean ± SEM (n = 3); ***p<0.001.

(S4A) Immunoprecipitation (IP) for LPAR1 in M2 cells expressing Myc-FLNA and immunoblot (IB) for Myc-FLNA, MRTF-A and LPAR1. BO: Dynabeads-only control, without antibody.

(S4B) Immunoblotting in HuH7 cells treated without (-), with 1 µM or 10 µM Calpain inhibitor III using FLNA and HSP90 antibodies.

(S4C) Representative pictures of senescence associated SA-β-galactosidase staining in HuH7 cells expressing negative control siRNA (sictrl) or FLNA siRNA (siFLNA), Myc-FLNA wt (FLNA wt), Myc-FLNA S2152A (FLNA S2152A) or -empty vector (EV) (left) or mCherry-FLNA wt (FLNA wt), mCherry‑FLNA Δ571‑866 (FLNA Δ571‑866) and –empty vector (EV) (right).

(S4D) Immunoblotting for mCherry-FLNA, FLNA and HSP90 in lysates of HuH7 cells transfected with FLNA siRNA (siFLNA) or negative control siRNA (sicrtl) and mCherry-FLNA wt (FLNA wt), mCherry‑FLNA Δ571‑866 (FLNA Δ571‑866) and –empty vector (EV) (right).
