# Supplementary material for: LPA receptor 1 (LPAR1) is a novel interaction partner of Filamin A that promotes Filamin A phosphorylation, MRTF-A transcriptional activity and oncogene-induced senescence
Source: Oncogenesis. 2022 Dec 28;11(1):69. doi: 10.1038/s41389-022-00445-z (PMC9797565; doi:10.1038/s41389-022-00445-z)
Supplement: Supplementary file 6 — Supplementary tables [file 41389_2022_445_MOESM6_ESM.pdf]

Supplementary Tables

Table S1: siRNA sequences used for transient knockdown

siRNA	sequence (sense and antisense)	manufacturer
siRNA negative control	5’-CGUACGCGGAAUACUUCGA[dt][dt]-3’<br>5’-UCGAAGUAUUCCGCGUACG[dt][dt]-3’	Sigma-Aldrich, Taufkirchen, Germany
siRNA LPAR1	ON-TARGETplus Human LPAR1 (1902) siRNA – SMARTpool	Horizon Discovery Cambridge, United Kingdom
siRNA LPAR2	ON-TARGETplus Human LPAR2 (9170) siRNA - SMARTpool	Horizon Discovery Cambridge, United Kingdom
siRNA LPAR3	ON-TARGETplus Human LPAR3 (23566) siRNA - SMARTpool,	Horizon Discovery Cambridge, United Kingdom
siRNA FLNA	5’-GCACAUGUUCCGUGUCCUA[dt][dt]-3’<br>5’-UAGGACACGGAACAUGUGC[dt][dt]-3’	Dharmacon, Lafayette, CO, USA

Table S2: plasmids used for transient transfection

plasmids	Provided	Published
dsRed-EV	Gift from Michael Davidson (addgene plasmid # 54493)	unpublished
Flag-EV	Sigma-Aldrich, Taufkirchen, Germany	-
Flag-MKL1	-	*Cen et al, Mol Cell Biol. 2003*
Myc-FLNA	Gift from John Blenis (Addgene plasmid # 8982)	*Woo et al, Moll Cell Biol. 2004*
Myc-FLNA S2152A	Gift from John Blenis (Addgene plasmid # 8983)	*Woo et al, Moll Cell Biol. 2004*
pcDNA3.1-Flag-p120-SH3	Gift from Reza Ahmadian, Heinrich-Heine-Universität Düsseldorf, Germany	-
mCherry-FLNA	Gift from Michael Davidson (addgene plasmid # 55047)	unpublished
mCherry-FLNA Δ571-866	descripted in material and methods	descripted in material and methods
HA-FLNA Mut.1-8	Gift from Fred Berry	*Kircher et al., Sci Signal. 2015*
Tango LPAR1	*Addgene* # 66418	-
p3xFLAG-MKL1	Addgene #119878	-
pECFPC MRTF-A	produced	-
dsRed FLNA S2152A	produced	-

Table S3: Primes sequences used for DNA cloning

primer	sequence
mCherry-FLNA Δ571-866 Fw<br>mCherry-FLNA Δ571-866 Rv	5’-GAG CCC TCT CAT GAC GCC-3’<br>5’-CAC CTT CAC TTC GAA GGG AC -3’

Table S4: Primer sequences used for qRT-PCR

primer	sequence
18S rRNA Fw<br>18S rRNA Rv	5’-TCG AGG CCC TGT AAT TGG AAT-3’<br>5’-CCC TCC AAT GGA TCC TCG TTA-3’
GLIPR1 Fw<br>GLIPR1 Rv	5’-TCT TTC CAA TGG AGC ACA TTT-3’<br>5’-TCT TAT ATG GCC AAG TTG GGT AA-3’
Ki67 Fw<br>Ki67 Rv	5´□TCA AGG ACC TGA TTC AGG AGA AG□3´<br>5´□GTG CAC TGA AGA ACA CAT TCC □3´
LPAR1 Fw<br>LPAR1 Rv	5’- CTG AAG ACT GTG GTC ATT GTG C□3´<br>5’- AAC CAC AGA GTG GTC ATT GCT G□3´
LPAR2 Fw<br>LPAR2 Rv	5’- GTC AAG ACT GTT GTC ATC ATC CT□3´<br>5’- GGA AGC ATG ATG CGA GTG CG□3´
LPAR3 Fw<br>LPAR3 Rv	5’- GATTGTTTTGTGTGTTGGGACG□3´<br>5’- TGGTCAGGTTGCTATGGACC□3´
SM22 Fw<br>SM22 Rv	5’-GGC CAA GGC TCT ACT GTC TG-3’<br>5’-CCC TTG TTG GCC ATG TCT-3’
SMA Fw<br>SMA Rv	5’□CCT ATC CCC GGG ACT AAG AC□3’<br>5’□AGG CAG TGC TGT CCT CTT CT□3’
SRF Fw<br>SRF Rv	5’-AGC ACA GAC CTC ACG CAG A-3’<br>5’-GTT GTG GGC ACG GAT GAC-3’

Table S5: Primary antibodies used for immunoblotting

antibody	manufacturer
anti-Actin (rabbit)	Merck, Darmstadt, Germany
anti-Erk1/2 (p44/42 MAPK) (rabbit)	Cell Signaling Technology, Danvers, MA, USA
anti-Flag (mouse)	Sigma-Aldrich, Taufkirchen, Germany

anti-FLNA (mouse)	Merck KGaA, Darmstadt, Germany
anti-FLNA pS2152 (rabbit)	Cell Signaling Technology, Danvers, MA, USA
anti-H3K9me3 (rabbit)	Actif Motif, Carlsbad, USA
anti-HA (mouse)	Santa Cruz Biotechnology, Santa Cruz, CA, USA
anti-HSP90 (mouse)	Santa Cruz Biotechnology, Inc., Dallas, TX, USA
anti-LPAR1 (mouse)	Santa Cruz Biotechnology, Inc., Dallas, TX, USA
anti-LPAR1 (rabbit)	Sigma-Aldrich, Taufkirchen, Germany
anti-LPAR1 (rabbit)	Abcam, Cambridge, UK
anti-mCherry (rabbit)	Abcam, Cambridge, UK
anti-MRTF-A (mouse)	Santa Cruz Biotechnology, Inc., Dallas, TX, USA
anti-MRTF-A (goat)	Santa Cruz Biotechnology, Inc., Dallas, TX, USA
anti-Myc (rabbit)	Cell Signaling Technology, Danvers, MA, USA
anti-Myc (mouse)	Cell Signaling Technology, Danvers, MA, USA
anti-Myoferlin (mouse)	Santa Cruz Biotechnology, Inc., Dallas, TX, USA
anti-phospho-p44/42 MAPK (Erk1/2) (Thr202/Tyr204) (rabbit)	Cell Signaling Technology, Danvers, MA, USA
anti-pRb (mouse)	BD Bioscience, San Jose, CA, USA
anti-SMA (rabbit)	Abcam, Cambridge, UK
anti-SRF (rabbit)	Santa Cruz Biotechnology, Inc., Dallas, TX, USA
anti-Transgelin (SM22) (mouse)	Merck, Darmstadt, Germany

Table S6: Secondary antibodies used for immunoblotting

antibody	manufacturer
anti-mouse IgG, HRP-linked antibody	Cell Signaling Technology, Danvers, MA, USA
anti-rabbit IgG, HRP-linked antibody	Cell Signaling Technology, Danvers, MA, USA

Table S7: Primary antibodies used for immunofluorescence and proximity ligation assay

antibody	manufacturer
anti-FLNA (mouse)	Merck KGaA, Darmstadt, Germany
anti-LPAR1 (mouse)	Santa Cruz Biotechnology, Inc., Dallas, TX, USA
anti-LPAR1 (rabbit)	Abcam, Cambridge, UK
anti-MKL1 (goat)	Santa Cruz Biotechnology, Santa Cruz, CA, USA
anti-MKL1 (mouse)	Santa Cruz Biotechnology, Inc., Dallas, TX, USA
anti-Phalloidin Alexa-488 conjugated	Invitrogen, Karlsruhe, Germany
anti-Paxillin (mouse)	BD Biosciences, Heidelberg, Germany
anti-mCherry (rabbit)	Abcam, Cambridge, UK
anti-Myc (mouse)	Cell Signaling Technology, Danvers, MA, USA

Table S8: secondary antibodies used for immunofluorescence

antibody	manufacturer
Donkey anti-mouse IgG, Alexa-488-linked antibody	Invitrogen, Karlsruhe, Germany
Donkey anti-goat IgG, Alexa-488-linked antibody	Invitrogen, Karlsruhe, Germany
Donkey anti-rabbit IgG, Alexa-488-linked antibody	Invitrogen, Karlsruhe, Germany
Donkey anti-mouse IgG, Alexa-647-linked antibody	Invitrogen, Karlsruhe, Germany

Table S9: Primary antibodies used for pulldown in immunoprecipitation

**antibody**	**manufacturer**
anti-Flag (mouse)	Sigma-Aldrich, Taufkirchen, Germany
anti-FLNA (mouse)	Merck KGaA, Darmstadt, Germany
anti-HA (mouse)	Santa Cruz Biotechnology, Santa Cruz, CA, USA
anti-LPAR1 (mouse)	Santa Cruz Biotechnology, Inc., Dallas, TX, USA
anti-LPAR1 (rabbit)	Abcam, Cambridge, UK
anti-mCherry (rabbit)	Abcam, Cambridge, UK
anti-Myc (mouse)	Cell Signaling Technology, Danvers, MA, USA
